# Persistent fear of aftershocks, impairment of working memory, and acute stress disorder predict post-traumatic stress disorder: 6-month follow-up of help seekers following the L’Aquila earthquake

**DOI:** 10.1186/2193-1801-2-636

**Published:** 2013-11-27

**Authors:** Rita Roncone, Laura Giusti, Monica Mazza, Valeria Bianchini, Donatella Ussorio, Rocco Pollice, Massimo Casacchia

**Affiliations:** Department of Life, Health and Environmental Sciences, Unit of Psychiatry, University of L’Aquila, L’Aquila, 67100 Italy

## Abstract

The aim of our 6-month follow-up study was to assess predictors of post-traumatic stress disorder (PTSD) among individuals seeking treatment at the General Hospital Psychiatric Unit within the first month following the L’Aquila earthquake. Clinical, trauma-related and neurocognitive variables were considered.

At the 6-month follow-up, 91 (74.5%) out of 122 subjects were re-assessed and administered the Impact of Events Scale-revised (IES-R) for the detection of PTSD according to DSM-IV criteria. Within 4 weeks following the earthquake, patients were assessed with a checklist of traumatic-event-related variables, along with the Stanford Acute Stress Disorder Questionnaire (SASDQ) for the detection of ASD, with a short battery on working (Wechler Memory Scale-R, Digit Forward and Backward) and verbal memory (subtest of Milan Overall Dementia Assessment, MODA).

A statistically significant higher proportion of subjects affected by ‘partial’ ASD showed a PTSD diagnosis (80.6%, N = 29) compared to not diagnosed subjects (40%, N = 22) and a PTSD diagnosis was shown by all the 4 subjects (4.4%) affected by ‘full’ ASD at the entry in the study. At the 6-month follow-up 56% of the sample could be considered affected by PTSD on the IES-R scale. The results of the logistic regression analysis on our selected predictors indicated that the persistent fear of aftershocks seemed to increase by over 57 times the likelihood of positive estimate of PTSD, followed by impairment of working memory backward (OR 48.2), and having being diagnosed as ASD case in the first 4 week after the earthquake (OR 17.4).

This study underlines the importance of identifying PTSD predictors, in order to planning early treatment interventions after natural disasters.

## Introduction

Natural disasters, such as earthquakes, expose people to distressing consequences. These can include physical injuries, impaired cognitive function, emotional dysregulation, loss of loved ones, damage and/or loss of property, and displacement (Armenian et al.^2000^; Basoglu et al.^2002^; Salcioglu et al.^2003^; Bland et al.^2005^; Chang et al.^2005^; Priebe et al.^2009^; Mazza et al.^2012^). A critical task is to distinguish trauma survivors who will suffer transient stress reactions from those who will experience stress reactions that will persist toward a long-term mental health disorder (Bryant^2011^).

Many factors have been identified that seem to impact the mental health of populations affected by natural disasters, which contribute to the onset and severity of posttraumatic stress disorder (PTSD): female gender (Carr et al.^1995^; Karanci and Rustemli^1995^; Sharan et al.^1996^; C. C. Chen et al.^2001^; Basoglu et al.^2002^; Livanou et al.^2002^; Salcioglu et al.^2003^; Kiliç and Ulusoy^2003^; Chang et al.^2005^; Montazeri et al.^2005^; Sattler et al.^2006^; Hansson et al.^1994^; C. H. Chen et al.^2007^; Kuwabara et al.^2008^; Priebe et al.^2009^; Steel et al.^2011^; Ahmad et al.^2010^; Dell'Osso et al.2011b; Zhang and Ho^2011^), older (Carr et al.^1997^; Salcioglu et al.^2003^; Toyabe et al.^2006^; C. H. Chen et al.^2007^; Priebe et al.^2009^; Zhang and Ho^2011^) or younger age at trauma (Kato et al.^1996^), lower education (Karanci and Rustemli^1995^; Armenian et al.^2000^; Basoglu et al.^2002^; Kiliç and Ulusoy^2003^; Montazeri et al.^2005^; C. H. Chen et al.^2007^; Priebe et al.^2009^; Wang et al.^2009^; Ahmad et al.^2010^), epigenetic vulnerability (Broekman et al.^2007^; Mehta and Binder^2012^), previous psychiatric illness (Nolen-Hoeksema and Morrow^1991^; Basoglu et al.^2002^; Salcioglu et al.^2003^), fear of death at the time of the disaster (Basoglu et al.^2002^; Livanou et al.^2002^; Bergiannaki et al.^2003^; Salcioglu et al.^2003^; Kiliç and Ulusoy^2003^; Basoglu et al.^2004^; Kuwabara et al.^2008^), degree of exposure to the disaster (Goenjian et al.^1994^; Carr et al.^1995^; Armenian et al.^2000^; Sattler et al.^2006^; Wang et al.^2009^; Zhang and Ho^2011^), closer proximity to the epicenter of an earthquake (Ahmad et al.^2010^), loss of loved ones (Basoglu et al.^2002^; Livanou et al.^2002^; Montazeri et al.^2005^; Goenjian et al.^1994^; Dell'Osso et al.2011a), property or resource loss (Freedy et al.^1994^; Armenian et al.^2000^; C. C. Chen et al.^2001^; Bland et al.^2005^; Sattler et al.^2006^; C. H. Chen et al.^2007^; Kuwabara et al.^2008^; Sharan et al.^1996^), having been trapped/injured under rubble and/or participation in rescue work (Basoglu et al.^2002^; Salcioglu et al.^2003^; Kuwabara et al.^2008^; Ehring et al.^2011^), greater number of traumatic experiences (Chang et al.^2005^), relocation after a disaster (Najarian et al.^2001^; Kiliç et al.^2006^), less social support (Bland et al.^1997^; Armenian et al.^2000^; Sattler et al.^2006^; Bland et al.^2005^; Wang et al.^2009^), and diminished individual coping (Olff et al.^2005^). Within one month after exposure to an extreme traumatic stressor, a mental disorder, such as acute stress disorder (ASD) can manifest. ASD is characterized by the development of dissociative and re-experiencing of symptoms, marked avoidance, anxiety or increased arousal, and significant distress or functional impairment. A diagnosis of ASD seems to have predictive power for PTSD by reducing the number of symptoms required within the dissociative cluster criteria, identifying subsyndromal ASD (Bryant^2011^).

Neuropsychological studies have documented that PTSD is associated with significant impairment in cognitive functioning, specifically attention, working memory, and verbal memory functions (Bremner et al.^1993^; Bremner et al.^1995^; Yehuda et al.^1995^; Vasterling et al.^1998^; Gilbertson et al.^2001^; Vasterling et al.^2002^; Brandes et al.^2002^; Yehuda et al.^2005^; Yehuda et al.^2004^; Johnsen and Asbjornsen^2008^; Lagarde et al.^2010^). Intrusive memories related to PTSD, and the elevated arousal levels of those memories, have been hypothesized to interfere with on-going cognitive processing. These intrusions are assumed to produce memory impairments in working memory and attentive functions (Clark et al.^2003^; Moores et al.^2008^) and verbal memory performance (Bremner et al.^1993^; Bremner et al.^1995^; Uddo et al.^1993^; Yehuda et al.^1995^; Vasterling et al.^1998^; Vasterling et al.^2002^; Brandes et al.^2002^; Johnsen and Asbjornsen^2008^).

However, most research in the field focuses on human-generated traumas, and few studies have investigated cognitive functions among survivors of natural trauma (Parslow and Jorm^2007^; Eren-Kocak et al.^2009^). In a recent study, earthquake survivors with diagnosed PTSD displayed diminished verbal memory and prefrontal functions (i.e., organization and monitoring functions within the frontal lobe related to verbal memory) than both individuals who had previously been diagnosed with PTSD and healthy traumatized subjects (Eren-Kocak et al.^2009^).

At 3.32 a.m. on April 6, 2009, central Italy was struck by a 6.3 magnitude earthquake. The earthquake caused serious damage to the 13th century town of L’Aquila, which is seated within the mountainous Abruzzo region. The earthquake also caused significant damage to several medieval hill villages in the surrounding areas, killing 309 residents, injuring over 1,600, and leaving approximately 70,000 people homeless (Casacchia et al.^2012^). Approximately 44,000 people found accommodations in tented camps close to their place of residence, and an additional 20,000 were housed in hotels on the Abruzzo Adriatic Sea coast. Others sought shelter with friends and relatives throughout Italy. The earthquake also caused serious damage to St. Salvatore General Hospital in L’Aquila, where our psychiatric inpatient unit and outpatient services were located. During a 6-month period post-earthquake, there were several aftershocks (22 shocks with a local magnitude of 4.0 or higher).

We conducted a recent study with survivors of the L’Aquila earthquake who subsequently sought treatment during the first month following the earthquake (Casacchia et al.^2013^). We found that despite the high level of psychological distress (GHQ-12 ≥ 20, cut-off value) among 65.6% of subjects, only 6 subjects (4.9%) were classified as affected by “full” ASD, whereas 48 subjects (39.3%) could be considered as “partial” ASD cases (Casacchia et al.^2013^). Partial cases were defined as showing at least one symptom within each DSM-IV criterion (scoring higher than a 3 on each Stanford Acute Stress Reaction Questionnaire “SASRQ” scale (Cardena et al.^2000^)).

The aim of our study was to first assess the predictive power of our ASD cases in the development of PTSD among those seeking help during the first 4 weeks post-earthquake. We expected that the subjects affected by full and partial ASD could develop PTSD. Furthermore we tested the hypotheses that being trapped under rubble and experiencing an injury, as shown in our previous study, would be the strongest predictor of PTSD among earthquake stressors (i.e., fear of death during the earthquake, the persistent fear of aftershocks, loss of loved ones and property, damage to the home, and disruption of the social network). On the basis of findings from other studies (Brandes et al.^2002^; Johnsen and Asbjornsen^2008^; Lagarde et al.^2010^; Eren-Kocak et al.^2009^; Parslow and Jorm^2007^) we also investigated whether neurocognitive variables, such as verbal and working memory, play a role in moderating responses to the first traumatic stress reaction. In other words, impaired verbal and working memory might increase the risk of developing PTSD.

## Methods

### Sample

During the first month after the earthquake (April 7 to May 6, 2009), we assessed 122 earthquake victims who sought help at our Psychiatric Unit (Casacchia et al.^2013^). All subjects gave written informed consent for inclusion in our follow-up sample. At a 6-month follow-up (September to October 2009) 91 subjects (74.5% of the initial sample) consented to being re-assessed for the presence of a PTSD.

### Measures

Socioeconomic and demographic characteristics (gender, age, education level, marital status, having children, occupation, previous traumatic events, and previous contact with Mental Health Services “MHS”) were recorded for all subjects in the sample.

### Earthquake trauma events checklist

Data regarding disaster experiences were obtained with the use of the ***Earthquake Check*-*List L*’*Aquila*(*ECLA*)**, a self-report 10-item tool developed by our research group immediately after the disaster (Casacchia et al.^2013^). The ECLA recorded the trauma events experienced or witnessed by the subjects in response to the earthquake. This measure consists of 10 yes or no questions (see Table 1).

**Table 1:** **Earthquake check list L**’**Aquila**, **ECLA frequency of trauma events witnessed or experienced by the earthquake by the subjects included** (**no**=**91**)

1. Direct experience of the earthquake (%)	100
2. Having been trapped/injured under rubble during the earthquake and in need to seek help to an emergency ward (%)	7.7
3. Death of relative (%)	8.8
4. During the earthquake feeling like “going” to die (%)	58.2
5. Persistent fear for aftershocks	65.9
6. Damaged property (%)	35.2
7. Accommodation after the earthquake (%)	
Displaced from home	49.5
-Camp tents in the town	34.1
-Individual tent - camper van	13.2
-House of friends	2.2
-Hotel at the Adriatic seaside	7.7
Not displaced	50.5
-Home	8.8
-Come back home (not resident in L’Aquila)	28.6
8. Distress for lack of privacy (%)	29.7
9. Lack of neighbor - friends in the new accommodation (%)	35.2
10. Job loss in the town (%)	39.6

### Post-traumatic stress disorder

At the 6-month follow-up, all subjects were asked to complete the **Impact of Events Scale-Revised**, which is one of the most widely used self-report measures in the field of traumatic stress (Creamer et al.^2003^; Weiss and Marmar^1997^). The IES-R consists of 22 items with a 5-point Likert-type scale ranging from 0 (not at all) to 4 (often). Three subscale scores can be obtained by summing the relevant item scores: Intrusion, Avoidance, and Hyperarousal. Furthermore, Creamer et al. (Creamer et al.^2003^) proposed that individuals with an average IES-R score greater than 1.5, or a total IES-R score over 33, could be classified as a “probable PTSD case”. These two cut-off points were used in the present study to identify PTSD cases.

#### Neurocognitive measures

Neurocognitive measures were administered in the first 4 weeks following the earthquake.

#### Verbal memory

We administered the verbal memory subset (“**The short history**”) of the Milan Overall Dementia Assessment (MODA) (Spinnler and Tognoni^1987^), which includes 16 units (score range 0–16). This test is composed of a short history read to the subject. After this first reading, an immediate recall assessment is conducted; at the end of this task, the story is repeated. After 10 minutes, without any prior instruction, the subject is asked to recall the history (deferred recall). Within our sample a cut-off score of 8 for immediate and deferred recall was used.

#### Working memory

All participants were administered the **Wechsler Memory Scale-Revised (WMS-R** (Wechsler^1987^) to assess working memory. The digit span subtest is composed of both a forward and a backward recitation condition. For the digits forward (scoring range 0–9, digit strings from 0 to 9) subtest, the subject is verbally presented with a string of numbers and asked to repeat the numbers in order. In the digits backward condition, the individual is instructed to repeat the presented strings in reverse order. The digit span score combines the total number of digit strings correctly repeated in both conditions. A digits forward score lower than 5, and a digits backward score lower than 4, was used as working memory cut-offs.

The project protocol used in this study was included in the Project “programma di Supporto Psicosociale Emergenza Sisma”, SPES (Psychosocial Support and Earthquake Emergency). It was approved on May 2009 and granted by the Italian Minister of Health though the Region Abruzzo, n. DG9/03, 4/15/2010. The study was approved by the University of L’Aquila ethics committee in compliance with the Helsinki Declaration. Participants gave written informed consent prior to participation in the study.

### Statistical analysis

Statistical analysis was performed using SPSS (version 16.0). For all analyses, a *p* value less than 0.05 (two-tailed) was used to determine statistical significance. Descriptive statistics were used for demographic, trauma-related, clinical, and neurocognitive characteristics of the study sample. Group comparisons were performed using independent *t*-tests for continuous variables and chi-square tests for categorical variables.

Diagnosis of “full” ASD (assessed by the SASRQ scale) was defined by subjects satisfying all DSM-IV criterion, while a diagnosis of “partial” ASD was determined by subjects who had at least one symptom within each criterion of the SASRQ subscales (scoring ≥ 3 on any symptom for dissociative cluster “Criterion B”, re-experiencing “Criterion C”, avoidance “Criterion D”, general reactivity and autonomic hyperactivation “Criterion E”, reduced functioning “Criterion F”) (Casacchia et al.^2013^). Due to the low prevalence of full ASD among our sample, we did not include this diagnosis in our statistical predictive model.

Regression analyses were conducted for identifying potential predictors of PTSD. Logistic regression was used to test one predictive model.

We included four blocks of variables. In step 1, sociodemographic data (gender, age group, school education, having children) were included as potential predictors. Age was coded into three categories (15–24 years, 25–59 years, 60 years and above). This categorization was based on the assumption that younger and older people might be more at risk for developing PTSD. Education was coded into two categories (8 years or less and more than 8 years of education). In step 2, traumatic stressful events (feeling like you were “going” to die during the earthquake, having been trapped/injured under rubble, death of a relative, persistent fear of aftershocks, damaged property, displacement, distress due to reduced privacy, lack of friends, and loss of work) were included as potential predictors and coded into 2 categories (no/yes). In step 3, we included data related to the subjects’ diagnosis of ‘partial’ ASD at beginning of the study (coded into 2 categories, yes/no) and previous contact with MHS (coded into 2 categories, no/yes).

In step 4, neurocognitive variables were included: verbal memory as assessed by the “short history” subtest (coded into 2 categories, impairment/no impairment), and working memory as assessed by WMS-R, forward (coded into 2 categories: impairment/no impairment) and backward (coded into 2 categories: impairment/no impairment) digit span. We conducted odds ratios with 95% confidence intervals for the logistic regression analysis.

## Results

The main sociodemographic characteristics of the study sample are reported in Table 2. A statistically higher proportion of women was not found both at the start of the study and during the follow-up assessment. The mean age of the follow-up sample was relatively young, and more than 45% were students (L’Aquila had a relatively large University with approximately 30,000 enrolled students; most of them were not residents of the city where they studied. After the earthquake, most of the students went back to their hometowns and were commuting to L’Aquila to attend classes organized in tents and, when possible, in open spaces). Approximately 35% of the subjects were married, and of these, approximately 16% had children. Nearly 7% of subjects reported previous, serious traumatic events. Nearly one-third of the sample had previous contact with Mental Health Services.

**Table 2:** **Socio**-**demographic characteristics of the sample** (**no**=**91**)

Variables	Population
Sex (%)	
Women	57.1
Men	42.9
Age (mean, SD)	35.7 ys (17.7)
Year range (%)	
15-24	36.3
25-59	52.7
60 and over	11
Education level (years, SD)	12.1 ys (3.7)
Marital status (%)	
Unmarried/single	65.9
Married	34.1
Having children (%)	16.5
Occupation (%)	
Full-time work	12.1
Par-time work	9.9
Unemployed	3.3
House-wife	11.0
Student	46.2
Retired	4.4
Missing	13.1
Previous contact with mental health	
Services, MHS (%)	31.9

At the time of the earthquake, 44 subjects (48.3%) were residents of L’Aquila, 22 subjects (24.1%) lived in different small villages near L’Aquila (Paganica, San Demetrio Ne’ Vestini, Campo Imperatore, Capestrano, Scoppito, and Montereale), and the remaining 27.6% of the sample were living in L’Aquila but were not residents of the town (8.8% within the province, 6.6% within the Abruzzo region, 12.2% within other Italian regions). Immediately after the earthquake, L’Aquila was evacuated; the population was displaced, and this was still the case 6 months later.

### Trauma events

Table 1 shows the results of 10 items from the ***ECLA*** within our follow-up sample. Upon entry into the study, among all subjects directly exposed to the earthquake, almost 60% reported feeling like they were “going to die”. More than 65% were still fearful of aftershocks. Eight subjects (7.7%) needed to seek help at the hospital for physical injury immediately after the earthquake, and eight subjects (8.8%) reported the death of a relative.

Almost half (49.5%) of the subjects within our sample were displaced from their homes. Approximately 35% were housed within camp tents (about 170 camp tents were installed by National Civil Protection), and the majority of these subjects complained about a loss of privacy. More than 10% of our sample organized temporary accommodations, individually, with camper vans and tents close to their damaged property. Only a minority (less than 9%) of subjects remained at their homes within small rural villages that were close to L’Aquila; however, these villages experienced were less damage. Approximately 30% of subjects (mainly students who were not residents of the town) went back home and were commuting; they had to come back to L’Aquila frequently for their studies. About 10% relocated with relatives and friends or to hotels on the Abruzzo Adriatic Sea coast.

Around 35% of the sample lost their usual relationships with neighbors and friends. More than 35% had “severe” property damage, and approximately 40% of the subjects lost their jobs within L’Aquila.

### Post-traumatic stress disorder

The mean overall IES-R of the sample was 38.4 (*SD* = 19.8), with mean scores on the intrusion, avoidance and hyperarousal subscales being 13.8 (*SD* = 7.5), 11.5 (*SD* = 6.6) and 13.3 (*SD* = 7.1), respectively. This indicated high levels of PTSD symptoms. The more distressing symptoms (mean scores higher than 1.5) reported were Hyperarousal (65.9%), Intrusion (58.2%), and Avoidance (44%). Based on DSM-IV criteria, 56% of the sample could be categorized as being affected by PTSD.

At the beginning of the study, 6 subjects (4.9%) were affected by “full” ASD, whereas 48 subjects (39.3%) were affected by “partial” ASD; this produced a total of 54 (45.2%) ASD cases.

At the 6-month follow-up, 4 (66% of the initial recruited sample) out of the 6 subjects diagnosed as affected by “full” ASD, and 36 (75%) out the 48 subjects affected by “partial” ASD were re-assessed. All 4 subjects affected by “full” ASD presented with a PTSD diagnosis as compared to the non-full ASD subjects (54.4%, N = 43; chi-square: 3.219; d.f. 1; p = 0.073). The small size of the full ASD group did not allow the chi-square test to reach statistical significance. However, a significantly higher proportion of partial ASD (80.6%, N = 29) presented with a PTSD diagnosis compared to non-diagnosed partial ASD (38.3%, N = 18; chi-square: 14.821; d.f. 1; p < 0.000).

### Neurocognitive variables

#### Verbal memory

The mean value of the MODA “short history” was 11.17 (*SD* = 2.9; range 5–16). 14.3% of the sample (n = 13) scored lower than the cut-off score of 8.

A significant difference was observed between ASD cases diagnosed subjects (*M* = 10.22, *SD* = 3.10) and non-ASD diagnosed subjects (*M* = 11.75, *SD* = 2.57) (t-test for independent samples: t = 2.332; p = 0.022).

A significant difference was observed between PTSD diagnosed subjects (*M* = 10.65, *SD* = 2.93) and non-PTSD diagnosed subjects (*M* = 12.10, *SD* = 2.80) (t-test for independent samples: t = 2.270; p = 0.026). Any statistically significant differences were found in relation to gender and age.

#### Working memory

For the total sample, mean forward digit span was 5.88 (*SD* = 1.30; range 3–9) and mean backward digit span was 3.85 (*SD* = 1.00; range 2–7). For the digit forward subtest, 12.1% of the sample scored below the cut-off of 5; for the backward digit span backward, 36.3% scored below the cut-off of 4.

Mean differences for both digit span subtests were observed for subjects defined as ASD cases compared with non-affected subjects upon entry into the study. This was also the case between subjects affected by PTSD and non-affected subjects at the 6-month follow-up. Any statistically significant differences were found in relation to gender. More subjects coded into the “old” age category (60 years and above) had impaired performance for both the forward (37.5% vs 11%; chi-square: 4.238; p = 0.037) and backward (75% vs 37%; chi-square: 4.315; p = 0.038) digit span subtests as compared to subjects within the other age categories.

### Predictors of PTSD

The predictive model shown in Table 3 is the result of the logistic regression analysis for predicting PTSD from the IES-R scale.

**Table 3:** **Distribution of socio**-**demographic**, **trauma**-**event and clinical and neurocognitive variables and results of the 4 blocks of the model** (**for categorial variables**, **the reference group is indicated**)

	Block 1					Block 2					Block 3					Block 4				
	B	OR	CI 95% L	CI 95% U	p	B	OR	CI 95% L	CI 95% U	p	B	OR	CI 95% L	CI 95% U	p	B	OR	CI 95% L	CI 95% U	p
**Gender**																				
Women/man	-0,8219	0,4396	0,16	1,206	0,11	-1,2625	0,2829	0,0644	1,243	0,0946	-1,327	0,265	0,0486	1,447	0,125	-2,254	0,105	0,010	1,063	0,056
**Age group**																				
Other/15-24	-0,6542	0,5198	0,173	1,561	0,243	-0,796	0,4511	0,0963	2,114	0,3124	-0,447	0,639	0,0964	4,24098	0,643	-0,851	0,427	0,046	3,962	0,454
Other 60 and over	-07695	0,4633	0,081	2,664	0,389	-1,1705	0,3102	0,0187	5,135	0,4137	-0,398	0,671	0,0377	11,9544	0,786	-2,127	0,119	0,000	36,207	0,466
**Education**																				
8 years or less/more than 8 years	-0,1161	0,8904	0,288	2,75	0,84	0,344	1,4107	0,2613	7,616	0,6892	0,2487	1,282	0,1764	9,31903	0,806	0,978	2,658	0,237	29,795	0,428
**Children**																				
No/yes	-1,3783	0,252	0,052	1,232	0,089	-1,5834	0,0191	2,205	2,205	0,1911	-1,916	0,147	0,0104	2,044	0,153	-3,722	0,024	0,000	2,102	0,102
**Feeling like “going” to die**																				
No/yes						0,096	1,1009	0,2601	4,659	0,8961	-0,033	0,968	0,1864	5,02403	0,969	-0,167	0,846	0,136	5,258	0,858
**Physical injuries**																				
No/yes						3,514	33,60	1,1013	1024,8	0,0439	2,1105	8,252	0,1796	379,055	0,280	-0,377	0,174	0,003	155,542	0,902
**Death of relative**																				
No/yes						1,681	5,3719	0,3338	86,455	0,2356	1,989	7,308	0.3552	150,384	0,197	2,577	13,153	0,421	411,151	0,142
**Persistent fear for aftershocks**																				
No/yes						2,837	17,064	2,9639	98,238	0,0015	3,1793	24,029	2,8911	199,709	0,003	4,054	57,606	3,577	927,695	0,004
**Damaged property**																				
No/yes						0,592	1,807	0,3596	9,079	0,4726	0,7042	2,022	0,362	11,2976	0,422	1,092	2,981	0,272	32,662	0,371
**Displacement**																				
No/yes						-1,0254	0,3587	0,0537	2,394	0,2897	-1,184	0,306	0,0345	2,716	0,288	-0,267	0,765	0,056	10,516	0,842
**Distress for lack of privacy**																				
No/yes						1,065	2,9018	0,5096	16,523	0,23	0,5866	1,798	0,264	12,245	0,549	0,731	2,077	0,199	21,697	0,541
**Lack of friends**																				
No/yes						-1,327	0,2653	0,0495	1,422	0,1214	-1,286	0,276	0,0385	1,983	0,201	-2,418	0,089	0,007	1,173	0,066
**Job loss in the town**																				
No/yes						-0,1011	0,9039	0,2239	3,649	0,8871	-0,753	0,471	0,0818	2,713	0,399	-2,418	0,089	0,007	1,173	0,066
**partial ASD**																				
No/yes											2,3979	11,001	1,6413	73,7308	0,013	2,861	17,472	1,860	164,154	0,012
**Previous contact with MHS**																				
No/yes											0,4241	1,528	0,247	9,455	0,648	-1,345	0,260	0,019	3,542	0,312
**MODA verbal memory**																				
Impairment/no impairment																2,520	12,429	0,807	191,352	0,071
**Working memory - forward**																				
Impairment/no impairment																0,414	1,512	0,024	95,635	0,845
**Working memory - backward**																				
Impairment/no impairment																3,878	48,329	1,900	1229,521	0,019

OR = Odds Ratio; CI = Confidence Interval; L = Lower; U = Upper.

Within the first step, none of the variables provided statistically significant predictive power. In step 2, the likelihood of a positive estimate of PTSD increased by more than 33 times if subjects had been trapped/injured under rubble and by more than 17 times if they felt persistent fear of aftershocks (Table 3). Having been trapped/injured under rubble lost its predictive value in the third step, which included the addition of subjects’ previous diagnosis of partial ASD and their personal history of psychopathology (previous contact with MHS). The addition of these variables increased the predictive power of “feeling persistent fear of aftershocks” by almost 25 times, and the likelihood of a positive estimate of PTSD increased by almost 11 times if subjects were diagnosed as a partial ASD at the beginning of the study.

In the fourth step, both variables included in the third step showed an important increase in their predictive power, and the likelihood of a positive estimate of PTSD for feeling persistent fear of aftershocks increased by almost 58%. Having been diagnosed as a partial ASD at the beginning of the study increased the likelihood of a positive identification of PTSD by 17%. Backward digit span impairment increased the likelihood of a positive identification of PTSD by more than 48% (Table 3). The values of Nagelkerke’s *r*^2^ for the four blocks within the model in Table 3 are 0.107 for step 1, 0.567 for step 2, 0.652 for step 3, and 0.737 for step 4, indicating that the variables related to earthquake-trauma history among the subjects accounted for the highest amount of variance (46%) in predicting PTSD.

## Discussion

We expected a high number of subjects affected by PTSD among our ASD cases. Our results suggest that among subjects seeking psychological support at 4 weeks following the earthquake and at the 6-month follow-up, a larger proportion of the partial ASD (almost 81%) were suffering from PTSD as compared to non-diagnosed ASD subjects (more than 38%). A PTSD diagnosis was determined for all 4 subjects (4.4%) affected by “full” ASD at the beginning of the study. At the 6-month follow-up, 56% of the sample was affected by PTSD as measured by the IES-R scale.

Having been diagnosed as affected by partial ASD was not the strongest predictor of PTSD. The most relevant predictor of PTSD was the persistent fear of aftershocks, followed by working memory impairment (backward digit span). The latter finding partially confirms our hypothesis, suggesting an important role of neurocognitive variables in PTSD. Working memory impairment, shown within the first 4-weeks following the traumatic event, seems to greatly increase the likelihood of a positive identification of PTSD while verbal memory did not seem to contribute to our prediction model. Earthquake-trauma history accounted for a large proportion of variance (46%) in detecting positive cases of PTSD, whereas sociodemographic variables explained 10%; previous diagnosis of partial ASD, contact with psychiatric services, and backward digit span each accounted both for 8.5% of variance in detecting positive PTSD cases.

In comparing our study to studies conducted in Italy, a lower PTSD rate (27.6%) was found 1 year following a landslide in Sarno, Southern Italy, in May 1998 (Catapano et al.^2001^). A lower rate (14.5%) was also observed 6 months after the 2002 earthquake in Molise (a rural region of Italy) within a large community sample (Priebe et al.^2009^). Among studies concerning the 2009 L’Aquila earthquake, a mental health survey, conducted between March and December 2010 on a total of 1,078 young subjects (*M* = 21.4, *SD* = 5.6 years; 8% of the population within the 16–30 age range and 1.5% of the general population), indicated a marked increase in substance abuse among survivors. Additionally, 16% of subjects were affected by PTSD (Pollice et al.^2011^). A much higher rate was observed in a study conducted on 512 students attending the last year of high school about 10 months after the L’Aquila earthquake. A full diagnosis of PTSD was detected in 37.5% of the students examined, with significantly higher rates in women than men; 29.9% of students reported partial PTSD (Dell'Osso et al.2011b). Our sample was a very selective sample of severely distressed individuals, which perhaps helped inflate the high prevalence of PTSD (greater than 55%). This highlights the marked variability in the prevalence of PTSD across studies, partially related to the heterogeneity of screening instruments (Bryant^2011^).

Among subjects who developed PTSD, all of those affected by “full” ASD and 80% of those affected by partial ASD criteria 6 months before were included, and only the partial ASD group was significantly more affected by PTSD than the non-diagnosed ASD subjects were. Consistent with other studies, we found that the sensitivity of subsyndromal ASD for PTSD detection is more effective than using full ASD criteria. This suggests, “that focusing on general posttraumatic stress symptoms, rather than the more restrictive requirement of dissociation, results in more people who eventually develop PTSD being identified in the acute phase” (Bryant^2011^; Casacchia et al.^2013^).

None of the sociodemographic (gender, age, educational level, having children) or clinical (previous contact with MHS) variables were predictive of PTSD. In this sample our data do not confirm that women are more vulnerable to PTSD despite the large number of studies reporting such findings (Carr et al.^1995^; Karanci and Rustemli^1995^; Sharan et al.^1996^; C. C. Chen et al.^2001^; Basoglu et al.^2002^; Livanou et al.^2002^; Salcioglu et al.^2003^; Kiliç and Ulusoy^2003^; Chang et al.^2005^; Montazeri et al.^2005^; Sattler et al.^2006^; Hansson et al.^1994^; C. H. Chen et al.^2007^; Kuwabara et al.^2008^; Priebe et al.^2009^; Steel et al.^2011^; Ahmad et al.^2010^; Dell'Osso et al.2011b; Zhang and Ho^2011^).

The persistent fear of aftershocks was highly predictive of PTSD, thereby confirming the results obtained from previous studies (Kuwabara et al.^2008^). Indeed, the epicentral region saw dozens of significant aftershocks following the main earthquake; this likely resulted in sustained psychological trauma for subjects who were already traumatized by the main earthquake.

Our hypothesis that PTSD would be predicted by fear of death at the time of the earthquake (Bergiannaki et al.^2003^; Basoglu et al.^2002^; Livanou et al.^2002^; Salcioglu et al.^2003^; Kiliç and Ulusoy^2003^; Basoglu et al.^2004^; Kuwabara et al.^2008^) was not confirmed. Additionally, our findings do not confirm the negative impact of property damage, object resource loss (Freedy et al.^1994^; Sharan et al.^1996^; Armenian et al.^2000^; C. C. Chen et al.^2001^; Bland et al.^2005^; Sattler et al.^2006^; C. H. Chen et al.^2007^; Kuwabara et al.^2008^), or displacement (Kiliç et al.^2011^; Kiliç et al.^2006^; Kiliç and Ulusoy^2003^; Najarian et al.^2001^; Najarian et al.^1996^) in predicting PTSD.

Nevertheless, certain earthquake-stressors explained a large portion of variance (46%) within our PTSD prediction model, while neurocognitive variables accounted for less than 10% of the predictive variability in PTSD.

Regarding our memory functions results, our findings are in agreement with previous studies showing cognitive abnormalities among PTSD sufferers (through human-enacted traumas) (Uddo et al.^1993^; Yehuda et al.^1995^; Clark et al.^2003^; Brandes et al.^2002^; Vasterling et al.^1998^; Samuelson et al.^2006^; Moores et al.^2008^; Johnsen and Asbjornsen^2008^; Lagarde et al.^2010^). We expected that both memory functions (verbal and working memory) would be impaired by the traumatic event and our study confirmed our hypothesis, since both verbal and working memory functions were impaired in our ASD cases and PTSD diagnosed subjects as compared to the non-diagnosed subjects. In our model, only the impairment of working memory (as measured by backward digit span) seemed to be predictive of developing PTSD, showing a 50% increase in the likelihood of such diagnosis, and confirming the findings of a recent study of earthquake survivors (Eren-Kocak et al.^2009^). In that study, memory dysfunction, and functioning within the prefrontal lobe, was interpreted as factors leading to vulnerability for developing PTSD.

To date, two hypothetical domains have been identified regarding cognitive impairment in PTSD, namely impaired verbal memory (Bremner et al.^1993^; Bremner et al.^1995^) and impaired attention and working memory (Uddo et al.^1993^; Vasterling et al.^1998^). Cognitive deficits in PTSD might reveal the involvement of specific brain areas with this disorder (Brandes et al.^2002^). Accordingly, impaired verbal memory has been attributed to hippocampal dysfunction that, in PTSD, might reflect a damaging effect of chronic stress on hippocampal cells (Bremner et al.^1995^; McEwen^2000^). Deficits in attention and working memory have been argued to require the involvement of the prefrontal lobe (Vasterling et al.^1998^). Our findings are in partial agreement with a study from Brandes et al. (Brandes et al.^2002^) showing that impaired working memory is associated with early PTSD symptoms. This suggests that such difficulties might affect the long-term recollection of traumatic events. Conversely, our findings regarding the significant relationship between PTSD symptoms and verbal memory are not in agreement with Brandes et al. (2002), the latter showing a lack of an association between 10-day early PTSD symptoms and verbal memory impairment. Their results suggest that impairment in verbal memory might develop during the course of chronic PTSD (Bremner et al.^1995^; Yehuda et al.^1995^). The lack of verbal memory impairment in our predictive model could be explained by our sample displaying a transient form of PTSD; while assessed 6 months later, the traumatic event might not have facilitated chronic PTSD by that point.

This study has four main limitations. First, our study cannot be compared to community-based epidemiological studies because our follow-up sample included only subjects who were seeking psychological support within the disaster area. The second limitation is the small size of our sample compared to the population of 70,000 people who were exposed to the earthquake. Several individuals were dislocated to different places; therefore, these people could not seek help from our psychiatric services. The third limitation could be ascribed to the use of self-report instruments (the SASRQ as a diagnostic tool for ASD, and the IES-R as a diagnostic tool for PTSD); however, the poor emergency conditions in which local mental health professionals were working immediately after the earthquake did not allow for the administration of clinical interviews. A final limitation concerns the “short” and limited neurocognitive battery used in our study, administered only at entry into the study.

## Conclusions

The early detection of psychological reactions to traumatic events, such as natural disasters, allows for the relief of the immediate distress and prevention of negative long-term effects on mental health. Our findings show that a higher proportion of subjects affected by full or partial ASD developed PTSD within a 6-month period following the traumatic event. On the basis of an earlier assessment, this study underlines the importance of identifying PTSD predictors, such as the persistent fear of aftershocks, working memory impairment, and posttraumatic stress symptoms (referred to as partial ASD).

The fact that memory disturbances do accompany acute PTSD diagnosis is especially relevant for clinical management. If not addressed by clinicians, it is likely that compromised neurocognitive functions might reduce a patient’s ability to cope with various obstacles during the posttraumatic phase, impede recovery, and increase the likelihood of developing chronic PTSD.

Pragmatically, psychological and cognitive difficulties in the early aftermath of a traumatic event should be acknowledged when planning early treatment interventions. Prospective studies are needed to evaluate the extent to which early psychological and cognitive difficulties interfere with the recovery from PTSD symptoms. Additionally, we need to verify the predictive value of early clinical manifestations following natural disasters.
